# Supplementary material for: Compression-based inference of network motif sets
Source: PLoS Comput Biol. 2024 Oct 10;20(10):e1012460. doi: 10.1371/journal.pcbi.1012460 (PMC11495616; doi:10.1371/journal.pcbi.1012460)
Supplement: S1 Text — (PDF) [file pcbi.1012460.s001.pdf]

S1 Text: Motif mining based on hypothesis testing

The prevailing approach to network motif mining involves counting or estimating the frequency of each graphlet [1], and comparing it to its frequency in random networks generated by a null model [2, 3]. Subgraphs that appear significantly more frequently in the empirical network than in the random networks are deemed motifs. We here briefly describe the approach and discuss several of its main limitations.

Limitations

Hypothesis-testing-based motif mining suffers from several fundamental statistical limitations. Each of these problems can make such inferences statistically unreliable.

Gaussian assumption. First, motifs are inferred based either on a Z-test or on direct estimation of p values from sampling of random networks. The former approach assumes Gaussian statistics under the null, which is often not a good approximation [4]. In the latter approach, it is only possible to evaluate p-values that are larger than $1/M$ where M is the number of random networks analyzed. This is computationally expensive and precludes the evaluation of low p values, which in turn makes it practically impossible to correct for multiple testing using standard approaches, such as the Bonferroni correction, which effectively decreases the significance threshold by a factor of the order of the number of tests.

Dependence on the choice of null model. Second, the appropriate null model is often not known [5–7] or it may be computationally unfeasible to sample it [6–8]. However, results may crucially depend on the choice of null model [5, 6], potentially leading to the inference of spurious motifs (see Figs 3A–3D in the main text).

Correlated motif counts. Third, the frequencies of different graphlets are not guaranteed to be independent, so one should account for these correlations when performing statistical testing [4]. Moreover, one should also account for these correlations in the null model to avoid inferring spurious motifs [2, 9]. Given a graphlet α, hypothesis-based motif inference qualifies α as a network motif in an empirical network G if its frequency $f_\alpha(G)$ in G is significantly greater than in an ensemble of random networks $\mathcal{G}_\theta$ sampled from a null model P_θ.

Method

For uniformly sampling simple random networks, we use the shuffling algorithms described in S5 Text. When the edge swapping procedures are ergodic and unbiased, they are guaranteed to uniformly sample the corresponding ensembles of random networks after a large enough number of swaps [3]. However, the mixing time, i.e., the number of swaps needed for the generated networks to be practically independent, is not known in general [3]. To ensure that correlations between randomized networks are not likely to influence results (i.e., we try to favor hypothesis-testing based methods as much as possible), we perform $100E$ successful edge swaps to generate each random network. This does not guarantee an absence of correlations, but we note that the number of swaps is larger than what is typically prescribed in the literature (for reference 0.2E edge-swaps were used to generate each random network in [2], 3E in [10] and 6E in [11]).

We utilize the typical normality assumption of the graphlet frequencies under the null and employ as test statistic the Z-score given by

$$Z_{\alpha,\theta}(G) = \frac{f_\alpha(G) - \mu_{\alpha,\theta}}{\sigma_{\alpha,\theta}}, \tag{S1.1}$$

where

$$\mu_{\alpha,\theta} = \frac{1}{|\mathcal{G}_\theta|} \sum_{G' \in \mathcal{G}_\theta} f_\alpha(G') \tag{S1.2}$$

and

$$\sigma^2_{\alpha,\theta} = \frac{1}{|\mathcal{G}_\theta| - 1} \sum_{G' \in \mathcal{G}_\theta} \left[f^2_\alpha(G') - \mu^2_{\alpha,\theta} \right]. \tag{S1.3}$$

In all experiments, the size of $\mathcal{G}_\theta$ is set to 100 and the significance threshold (nominal alpha-level) is fixed at 0.01. To correct for multiple testing (one test for each graphlet), we employ a Bonferroni correction, which multiplies the raw p-values obtained directly from the Z-scores by $|\Gamma| \approx 10^4$. As displayed in Figs 3A–D, depending on the choice of the null, a considerable number of motifs can be falsely detected. A similar effect can also be seen in empirical data, where the number of motifs found varies enormously with the choice of null model (see S1 Fig), even though we corrected for multiple testing with the maximally conservative Bonferroni correction. S1 Fig also demonstrates that the motifs found vary significantly depending on the null, and that the smallest number of motifs is not necessarily found under the most restricted null hypothesis.

References

1. Pržulj N. Biological network comparison using graphlet degree distribution. Bioinformatics. 2007;23(2):e177–e183. doi:10.1093/bioinformatics/btl301.

2. Milo R, Shen-Orr S, Itzkovitz S, Kashtan N, Chklovskii D, Alon U. Network Motifs: Simple Building Blocks of Complex Networks. Science. 2002;298(5594):824–827. doi:10.1126/science.298.5594.824.

3. Fosdick BK, Larremore DB, Nishimura J, Ugander J. Configuring Random Graph Models with Fixed Degree Sequences. SIAM Rev. 2018;60(2):315–355. doi:10.1137/16M1087175.

4. Fodor J, Brand M, Stones RJ, Buckle AM. Intrinsic limitations in mainstream methods of identifying network motifs in biology. BMC Bioinformatics. 2020;21(1):165. doi:10.1186/s12859-020-3441-x.

5. Artzy-Randrup Y, Fleishman SJ, Ben-Tal N, Stone L. Comment on "Network Motifs: Simple Building Blocks of Complex Networks" and "Superfamilies of Evolved and Designed Networks". Science. 2004;305(5687):1107–1107. doi:10.1126/science.1099334.

6. Beber ME, Fretter C, Jain S, Sonnenschein N, Müller-Hannemann M, Hütt MT. Artefacts in statistical analyses of network motifs: general framework and application to metabolic networks. Journal of The Royal Society Interface. 2012;9(77):3426–3435. doi:10.1098/rsif.2012.0490.

7. Orsini C, Dankulov MM, Colomer-de Simón P, Jamakovic A, Mahadevan P, Vahdat A, et al. Quantifying randomness in real networks. Nat Commun. 2015;6(1):1–10. doi:10.1038/ncomms9627.

8. Ginoza R, Mugler A. Network motifs come in sets: Correlations in the randomization process. Phys Rev E. 2010;82(1):011921. doi:10.1103/PhysRevE.82.011921.

9. Stivala A, Lomi A. Testing biological network motif significance with exponential random graph models. Appl Netw Sci. 2021;6(1):1–27. doi:10.1007/s41109-021-00434-y.

10. Wernicke S, Rasche F. FANMOD: a tool for fast network motif detection. Bioinformatics. 2006;22(9):1152–1153. doi:10.1093/bioinformatics/btl038.

11. Ribeiro P, Silva F. g-tries: an efficient data structure for discovering network motifs. In: Proceedings of the 2010 ACM Symposium on Applied Computing. SAC '10. Sierre, Switzerland: Association for Computing Machinery; 2010. p. 1559–1566.
