# Supplementary material for: Compression-based inference of network motif sets
Source: PLoS Comput Biol. 2024 Oct 10;20(10):e1012460. doi: 10.1371/journal.pcbi.1012460 (PMC11495616; doi:10.1371/journal.pcbi.1012460)
Supplement: S2 Text — Table A in S2 Text recapitulates the computational costs for the motif-based inference when the maximal subgraph size is 4. Table B in S2 Text recapitulates the same costs when the maximal subgraph size is 5. (PDF) [file pcbi.1012460.s002.pdf]

S2 Text: Computation time & memory of the motif-based inference

Any motif mining algorithm, depending on its parameters and on the size and density of the input graph, is likely to suffer from a high computational cost. The main practical limit for our method is the consumption of hard memory and RAM, while for hypothesis testing-based method the limiting factor is typically the time complexity. As developed in S3 Text and Algorithm 2 in the "Methods" section, induced subgraphs, which ranges from 3 to 5 nodes, are listed and saved on the hard memory. If the subgraph maximal size is decreased to 4, the memory cost is considerably reduced. To give a sense of the applicability of our method in practice, we review the time and memory costs for the motif-based inference in two settings: (i) The saved induced subgraphs' sizes ranges from 3 to 4 nodes (Table **A**); the subgraph census and inference are performed on a cluster, using 4 CPUs, with single-thread parallelization; this condition mimics what can be done locally, on a common laptop. (ii) The saved induced subgraphs' sizes ranges from 3 to 5 nodes (Table **B**); the subgraph census and inference are performed on a cluster, using 10 CPUs, with single-thread parallelization.

Species	Connectome	Census—Memory (MB)	Census—Time (s)	Inference—Time (s)
C. elegans	Hermaphrodite—nervous system	25	1.049	8.918
C. elegans	Hermaphrodite—whole animal	44	1.028	14.971
C. elegans	Male—whole animal	37	0.968	14.435
Drosophila	Larva—Left MB	98	1.843	9.94
Drosophila	Larva—Right MB	95	1.725	8.817
Drosophila	Larva—Left & right MBs	713	8.288	54.774
C. intestinalis	Larva—whole animal	20	2.089	5.349

Table A: **Time and space costs for the inference of motif sets, with motif sizes ranging from 3 to 4 nodes.** The memory required and the temporal duration of the subgraph census are measured for connectomes that are compressible using our motif-based scheme. Contrary to the hypothesis testing-based approaches, the subgraph census is performed only once, instead of hundreds of times. However, a single subgraph census takes longer as the node labels of each induced subgraph are saved. In the case of small connectomes which have up to 400 neurons, the census can be handled by common laptops. Inference was repeated a hundred times, and we indicate in the rightmost column the duration corresponding to inference of the optimal model.

Species	Connectome	Census—Memory (GB)	Census—Time (s)	Inference—Time (s)
C. elegans	Hermaphrodite—nervous system	0.93	120.982	403.47
C. elegans	Hermaphrodite—whole animal	1.8	70.739	339.03
C. elegans	Male—whole animal	1.3	120.982	271.97
Drosophila	Larva—Left MB	3.7	138.685	590.75
Drosophila	Larva—Right MB	3.8	129.007	673.64
Drosophila	Larva—Left & right MBs	50	1424.428	3 468.60
C. intestinalis	Larva—whole animal	0.044	17.764	117.61
Drosophila	Larva—whole brain	21 (1%)	435.504	4 604.81
Drosophila	Adult—right MB	1300 (1%)	3 501.565	15 039.00

Table B: **Time and space costs for the inference of motif sets, with motif sizes ranging from 3 to 5 nodes.** The memory required and the duration of the subgraph census are measured for connectomes that are compressible using our motif-based scheme. With such large volumes of subgraph files, common laptops cannot handle the computational task. In this setting, we recommend storing subgraphs and running the inference algorithm on a HPC cluster. The last two lines confirm even more the amplitude of the demanding resources. For the two largest connectomes, we performed a stochastic subgraph census, where only 1% of the 5-node subgraphs are saved and only 10% of the 4-node subgraphs—overall, approximately 1% of the total number of subgraphs. Even if we remove the 1% of the 5-node subgraphs, the inference time complexity is still too great for common laptops.
