# Supplementary material for: Compression-based inference of network motif sets
Source: PLoS Comput Biol. 2024 Oct 10;20(10):e1012460. doi: 10.1371/journal.pcbi.1012460 (PMC11495616; doi:10.1371/journal.pcbi.1012460)
Supplement: S3 Text — (PDF) [file pcbi.1012460.s003.pdf]

S3 Text: Subgraph census: dealing with lists of induced subgraphs

Writing graphlet occurrences

Subgraph-census is a computationally hard task since it involves repeatedly solving the subgraph isomorphism problem. Since our algorithm uses not only the number of occurrences of each graphlet but also their placement in the original graph, we need not only to determine their number of occurrences but to list all weakly connected subgraphs from three to five nodes. There are about 10 000 distinct five-node graphlets, thus their frequencies can easily be stored on any modern laptop. However, the exhaustive lists of all graphlet occurrences can be very large, depending on the size and density of the network at hand. In the case of the brain regions of the adult *Drosophila melanogaster*, the magnitude of such connectomes is of the order of a thousand neurons, which, for their specific density, leads to at least several billion five-node subgraphs. In this case it is not possible to dynamically store all subgraph occurrences. Instead, we progressively write them to disk directly using textfile pointers thanks to the `ifstream` object of the C++ standard library (cplusplus.com/reference/fstream/ifstream). Each text file corresponds to a graphlet, containing isomorphic subgraphs divided on each line, with grouped node labels stored in CSV format.

Reading graphlet occurrences

Uniformly sampling subgraphs is part of our stochastic greedy algorithm. Randomizing the collected subgraph lists followed by sequential reading of their elements would be the most direct and simple approach to do this. Since it is not possible to store every induced subgraph in memory, we store the pointer positions of every line (i.e., every subgraph memory address) in a vector. These vector elements are shuffled and then read sequentially to perform a uniform sampling of the subgraphs. The memory gain, per graphlet textfile, is of the order of the textfile size times the graphlet size. In large connectomes, this memory gain is however not enough and it is not possible to store all subgraph pointers in memory. For this case, we implemented a procedure that divides the reading and shuffling of a graphlet textfile in chunks of a fixed size. If the number of subgraphs in a graphlet textfile (i.e., its number of lines) is lower than the chunk size, then the sampling is performed as described above. When the graphlet textfile is larger than the chunk size, the subgraph sampling is not uniform. Indeed, the initial node labeling of the input graph, together with the order of the subgraph mining imposed by Wernicke's algorithm, requires having access to all the listed subgraphs for a sampling to be exactly uniform. The larger the chunk reading size is, the less biased the sampling will be. For all the adult *Drosophila melanogaster* connectomes, we fixed the chunk size to a million subgraphs, so that the maximum number of stored subgraph pointers in RAM per graphlet is a million. When all the subgraphs of the current chunk of the full graphlet textfile are forbidden by the non-overlapping supernode constraint (see Algorithm 2 in the "Methods" section), another chunk is read.
