# Supplementary material for: Compression-based inference of network motif sets
Source: PLoS Comput Biol. 2024 Oct 10;20(10):e1012460. doi: 10.1371/journal.pcbi.1012460 (PMC11495616; doi:10.1371/journal.pcbi.1012460)
Supplement: S4 Text — Table A in S4 Text lists the parameters of the dyadic graph models and some important relationships between them. (PDF) [file pcbi.1012460.s004.pdf]

S4 Text: Graph codelengths and subgraph contraction costs

A Graph codelengths

In this supplementary note, we review the dyadic graph models used as base and null models, namely the Erdős-Rényi model (ER), the configuration model (CM), the reciprocal Erdős-Rényi model (RER), and the reciprocal configuration model (RCM). Model parameters, as well as and their relationships, are detailed throughout the note, and summarized in Table **A**.

Table A: Dyadic graph model parameters and simple identities.

Network feature	Notation	Expression
Number of directed edges	E	$\sum_{ij} A_{ij} = \sum_i k_i^+ = \sum_i k_i^- = E_d + 2E_m$
Out-degree of node i	k_i^+	$\sum_j A_{ij}$
In-degree of node i	k_i^-	$\sum_j A_{ji}$
Number of reciprocal (mutual) edges	E_m	$1/2 \sum_{i<j}(A_{ij} - A_{ji} + \lvert A_{ij} - A_{ji}\rvert) = 1/2 \sum_i \kappa_i^m$
Number of non-reciprocated (directed) edges	E_d	$1/2 \sum_{ij}(A_{ij} + A_{ji} - \lvert A_{ij} - A_{ji}\rvert) = \sum_i \kappa_i^+ = \sum_i \kappa_i^-$
Reciprocal (mutual) degree of node i	κ_i^m	$1/2 \sum_j (A_{ij} + A_{ji} - \lvert A_{ij} - A_{ji}\rvert)$
Non-reciprocated out-degree of node i	κ_i^+	$1/2 \sum_j (A_{ij} - A_{ji} + \lvert A_{ij} - A_{ji}\rvert) = k_i^+ - \kappa_i^m$
Non-reciprocated in-degree of node i	κ_i^-	$1/2 \sum_j (A_{ji} - A_{ij} + \lvert A_{ji} - A_{ij}\rvert) = k_i^- - \kappa_i^m$

A.1 Erdős-Rényi model (ER)

Multigraphs. The microcanonical Erdős-Rényi (ER) model encodes a multigraph G with a fixed number of nodes, N, and edges, E. The microcanonical probability distribution over the space of directed loop-free multigraphs is given by [1]

$$P_{(N,E)}(G) = \frac{E!}{\prod_i \prod_{j\neq i} A_{ij}!} [N(N-1)]^{-E}. \tag{S4.1}$$

The second factor in Eq. (S4.1) is the number of ways to place each edge between the $N(N-1)$ pairs of nodes, and the first factor accounts for the indistinguishability of the ordering of the multiedges. This leads to an entropy of

$$S_{(N,E)}(H) = E \log[N(N-1)] - \log E! + \sum_i \sum_{j\neq i} \log A_{ij}!. \tag{S4.2}$$

Simple graphs. The entropy of the simple, directed Erdős-Rényi model is found by counting the number of ways to place E edges amongst $N(N-1)$ pairs of nodes without overlap. This leads to

$$S_{(N,E)}(G) = \log \binom{N(N-1)}{E} = \log \frac{[N(N-1)]!}{[N(N-1)-E]!E!}. \tag{S4.3}$$

Model complexity. The parametric complexity of the ER model requires encoding the two positive integers that are its parameters. We make use of the standard codelength function for encoding natural numbers [2]. To a positive integer $n \in \mathbb{N}$, we attribute the cost

$$L_{\mathbb{N}}(n) = \log n(n+1). \tag{S4.4}$$

This leads to a codelength for describing (N, E) of

$$L(N,E) = L_{\mathbb{N}}(N) + L_{\mathbb{N}}(E). \tag{S4.5}$$

A.2 Configuration model (CM)

Multigraphs. The configuration model (CM) generates random networks with fixed in- and out-degrees of each node, i.e., the sequences $\mathbf{k}^+ = (k_i^+)$ and $\mathbf{k}^- = (k_i^-)$. The in-degree corresponds to the number of edges pointing towards the node, $k_i^- = \sum_j A_{ji}$, whereas the out-degree is the number of edges originating at the node, $k_i^+ = \sum_j A_{ij}$. The entropy of the configuration model is given by [3]

$$S_{(\mathbf{k}^+,\mathbf{k}^-)}(G) = \log E! - \sum_i \left(\log k_i^+! + \log k_i^-! - \sum_{j \neq i} A_{ij}! \right). \tag{S4.6}$$

Simple graphs. There are no exact closed-form expressions for the microcanonical entropy of the configuration model for simple graphs. We thus use the approximation developed in [4], which provides a good approximation for sparse graphs,

$$S_{(\mathbf{k}^+,\mathbf{k}^-)}(G) \approx \log \frac{E!}{\prod_i k_i^+! k_i^-!} - \frac{1}{2\ln 2} \frac{\langle {k_i^+}^2 \rangle \langle {k_i^-}^2 \rangle}{\langle k_i^+ \rangle \langle k_i^- \rangle}. \tag{S4.7}$$

Model complexity. Contrary to the Erdős-Rényi model, the configuration model is a *microscopic* description in the sense that it introduces two parameters per node (in addition to the number of nodes N) and thus a total of $2N+1$ parameters (as compared to 2 parameters for the ER model). Thus, while its entropy is always smaller than that of the ER model, its parametric complexity is higher. We consider two possible ways to encode the degree sequences $\mathbf{k}^+$ and $\mathbf{k}^-$. The simplest and most direct approach to encode a sequence $\mathbf{k}$ is to consider each element individually as a priori uniformly distributed in the interval of integers between $\delta = \min\{k_i \in \mathbf{k}\}$ and $\Delta = \max\{k_i \in \mathbf{k}\}$. This leads to a codelength of

$$L_U(\mathbf{k}) = N \log(\Delta - \delta + 1) + L_{\mathbb{N}}(\delta) + L_{\mathbb{N}}(\Delta). \tag{S4.8}$$

Assuming that the degrees are generated according to the same unknown probability distribution, it is typically more efficient to use a so called *plug-in* code [5,6], which describes them as sampled from a Dirichlet-multinomial distribution over the integers between δ and Δ. To each possible value $\delta \leq \mu \leq \Delta$ that a degree may take, we calculate the frequency r_μ of the value μ in $\mathbf{k}$. We then have

$$P_{\boldsymbol{\lambda}}(\mathbf{k}) = \frac{\Gamma(\Lambda)}{\Gamma(N+\Lambda)} \prod_{\delta \leq \mu \leq \Delta} \frac{\Gamma(r_\mu + \lambda_\mu)}{\Gamma(\lambda_\mu)}, \tag{S4.9}$$

where λ_μ are prior hyperparameters and $\Lambda = \sum_\mu \lambda_\mu$. When all $\lambda_\mu = \lambda = 1$, the priors are uniform probability distributions, while the case $\lambda_\mu = \frac{1}{2}$ corresponds to the Jeffreys prior [5]. The plug-in codelength is thus given by

$$L_{\boldsymbol{\lambda}}(\mathbf{k}) = -\log P_{\boldsymbol{\lambda}}(\mathbf{k}) + L_{\mathbb{N}}(\delta) + L_{\mathbb{N}}(\Delta). \tag{S4.10}$$

In the implementation of our algorithm, we select the encoding of the degree sequences among $L_U(\mathbf{k})$, $L_{\lambda=1}(\mathbf{k})$ and $L_{\lambda=1/2}(\mathbf{k})$ that results in the minimal codelength. Encoding this choice takes $\log 3$ bits. Including also the encoding of the number of nodes, N, the cost of encoding a degree sequence is

$$L_{\text{seq}}(\mathbf{k}) = \min\{L_U(\mathbf{k}), L_{\lambda=1}(\mathbf{k}), L_{\lambda=1/2}(\mathbf{k})\} + \log 3 + L_{\mathbb{N}}(N). \tag{S4.11}$$

Finally, the total parametric codelength of the configuration model is the sum of two sequence codelengths, leading to

$$L_{\text{seq}}(\mathbf{k}^+, \mathbf{k}^-) = L_{\text{seq}}(\mathbf{k}^+) + L_{\text{seq}}(\mathbf{k}^-). \tag{S4.12}$$

A.3 Reciprocal models

Reciprocated (or *mutual*) edges are an important feature of many biological networks [7–11]. Reciprocal edges confer to a network a partially symmetric structure. If they represent an important fraction of the total number of edges, this regularity can be used to significantly compress the network. To account for reciprocal edges in a simple manner, we consider them as a different edge type that are placed independently of directed edges. Thus, we model a multigraph G as the overlay of independent symmetric and asymmetric multigraphs, G^{sym} and G^{asym}, respectively, where G^{sym}

is an undirected multigraph and G^{asym} is a directed multigraph. The adjacency matrix of G is given by $\mathbf{A}(G) = \mathbf{A}(G^{\mathrm{sym}}) + \mathbf{A}(G^{\mathrm{asym}})$, and a reciprocal model's likelihood is equal to the product of the likelihoods of the symmetric and asymmetric parts, leading to a codelength of

$$L(G, \phi) = L(G^{\mathrm{sym}}, \phi^{\mathrm{sym}}) + L(G^{\mathrm{asym}}, \phi^{\mathrm{asym}}), \tag{S4.13}$$

where $\phi = (\phi^{\mathrm{sym}}, \phi^{\mathrm{asym}})$ and ϕ^{sym} and ϕ^{asym} are the parameters of the models used to encode the symmetric and asymmetric edges of G, respectively. In practice, we set for each pair $(i, j) \in \mathcal{N}(G) \times \mathcal{N}(G)$ the entries of the symmetric and asymmetric adjacency matrices to be

$$A_{ij}^{\mathrm{asym}} = \max(A_{ij} - A_{ji}, 0) = \frac{1}{2}(A_{ij} - A_{ji} + |A_{ij} - A_{ji}|), \tag{S4.14}$$

$$A_{ij}^{\mathrm{sym}} = \min(A_{ij}, A_{ji}) \qquad = \frac{1}{2}(A_{ij} + A_{ji} - |A_{ij} - A_{ji}|). \tag{S4.15}$$

This maximizes the number of edges in the symmetric representation, which minimizes the codelength since the entropy of an undirected model is lower than its directed counterpart and since each reciprocal edge encoded in G^{sym} corresponds to two directed edges.

A.4 Reciprocal Erdős-Rényi model (RER)

Multigraphs. The reciprocal version of the Erdős-Rényi model (RER) has 3 parameters, (N, E_m, E_d), where E_m is the number of reciprocal (mutual) edges and E_d is the number of directed edges, and we have $E = 2E_m + E_d$. The model's codelength is

$$L(G, (N, E_m, E_d)) = S_{(N,E_d)}(G^{\mathrm{asym}}) + S_{(N,E_m)}(G^{\mathrm{sym}}) + L(N, E_m, E_d). \tag{S4.16}$$

The entropy of the directed graph model, $S_{(N,E_d)}(G^{\mathrm{asym}})$, is given by Eq. (S4.2) with E replaced by E_d. The entropy of the symmetric part is given by [1]

$$S_{(N,E_m)}(G^{\mathrm{sym}}) = E_m \log[N(N-1)/2] - \log E_m! + \sum_i \sum_{i<j} \log A_{ij}^{\mathrm{sym}}!, \tag{S4.17}$$

Simple graphs. Contrary to multigraphs, the placement of directed and reciprocal edges is not entirely independent for simple graphs since we do not allow the edges to overlap. However, we can model the placement of one type of edges (say reciprocal edges) as being entirely random and the second type (e.g., directed) as being placed randomly between the pairs of nodes not already covered by the first type. This leads to a number of possible configurations of

$$\Omega_{(N,E_m,E_d)} = \binom{N(N-1)/2}{E_m}\binom{N(N-1)/2 - E_m}{E_d}, 2^{E_d}, \tag{S4.18}$$

where the first factor is the number of ways to place the reciprocal edges, the second factor is the number of ways to place the directed edges amongst the remaining node pairs without accounting for their direction, and the third factor is the number of ways to orient the directed edges. Simplifying and taking the logarithm yields the following expression for the entropy of the reciprocal ER model,

$$S_{(N,E_m,E_d)}(G) = \log \frac{[N(N-1)/2]!}{[N(N-1)/2 - E_m - E_d]! E_m! E_d!} + E_d. \tag{S4.19}$$

Model complexity. The RER model's parametric complexity is equal to

$$L(N, E_m, E_d) = L_{\mathbb{N}}(N) + L_{\mathbb{N}}(E_d) + L_{\mathbb{N}}(E_m). \tag{S4.20}$$

A.5 Reciprocal configuration model (RCM)

Multigraphs. Similarly to the ER model, we extend the configuration model to a reciprocal version (RCM) by introducing a third degree sequence, describing each node's *mutual* degree, defined as the number of reciprocal edges it partakes in. The model is thus defined by the set of parameters $(\boldsymbol{\kappa}^m, \boldsymbol{\kappa}^+, \boldsymbol{\kappa}^-)$ where $\kappa_i^m = \sum_j A_{ij}(G^{\mathrm{sym}})$ is the mutual degree of node i, $\kappa_i^+ = \sum_j A_{ij}(G^{\mathrm{asym}})$ is the non-reciprocated out-degree, and $\kappa_i^- = \sum_j A_{ji}(G^{\mathrm{asym}})$ is the non-reciprocated in-degree. The codelength of the reciprocal configuration model is equal to

$$L(G, (\boldsymbol{\kappa}^m, \boldsymbol{\kappa}^+, \boldsymbol{\kappa}^-)) = S_{(\boldsymbol{\kappa}^+,\boldsymbol{\kappa}^-)}(G^{\mathrm{asym}}) + S_{\boldsymbol{\kappa}^m}(G^{\mathrm{sym}}) + L(\boldsymbol{\kappa}^m, \boldsymbol{\kappa}^+, \boldsymbol{\kappa}^-). \tag{S4.21}$$

The entropy of the asymmetric graph is given by Eq. (S4.6) with $(\mathbf{k}^+, \mathbf{k}^-)$ replaced by $(\boldsymbol{\kappa}^+, \boldsymbol{\kappa}^-)$. The entropy of the symmetric graph is given by [3]

$$S_{\boldsymbol{\kappa}^m}(G^{\mathrm{sym}}) = \log(2E_m)! - \log(2E_m)!! - \sum_i \left(\log \kappa_i^m! - \sum_{j \neq i} A_{ij}^{\mathrm{sym}}! \right). \tag{S4.22}$$

Simple graphs. To derive an approximation for the entropy of the reciprocal configuration model for simple graphs, we follow the same approach as in [4] but with the three-degree sequences $(\boldsymbol{\kappa}^m, \boldsymbol{\kappa}^+, \boldsymbol{\kappa}^-)$ constrained instead of only two (see the subsequent Section B for a detailed derivation). This leads to a microcanonical entropy of

$$\begin{aligned} S_{(\boldsymbol{\kappa}^m,\boldsymbol{\kappa}^+,\boldsymbol{\kappa}^-)}(G) &\approx \log \frac{(2E_m)!!}{\prod_i \kappa_i^m!} + \log \frac{E_d!}{\prod_i \kappa_i^+! \kappa_i^-!} \\ &- \frac{1}{2\ln 2} \left(\frac{1}{2} \frac{\left\langle (\kappa_i^m)^2 \right\rangle^2}{\langle \kappa_i^m \rangle^2} + \frac{\left\langle \kappa_i^{+2} \right\rangle \left\langle \kappa_i^{-2} \right\rangle}{\left\langle \kappa_i^+ \right\rangle \left\langle \kappa_i^- \right\rangle} + \frac{\left\langle \kappa_i^+ \kappa_i^- \right\rangle^2}{\left\langle \kappa_i^+ \right\rangle \left\langle \kappa_i^- \right\rangle} + \frac{\left\langle \kappa_i^m \kappa_i^+ \right\rangle \left\langle \kappa_i^m \kappa_i^- \right\rangle}{\left\langle \kappa_i^m \right\rangle \left\langle \kappa_i^+ \right\rangle} \right). \end{aligned} \tag{S4.23}$$

Model complexity. The parametric part of the codelength is equal to

$$L(\boldsymbol{\kappa}^m, \boldsymbol{\kappa}^+, \boldsymbol{\kappa}^-) = L_{\mathrm{seq}}(\boldsymbol{\kappa}^+) + L_{\mathrm{seq}}(\boldsymbol{\kappa}^-) + L_{\mathrm{seq}}(\boldsymbol{\kappa}^m), \tag{S4.24}$$

with $L_{mathrmseq}$ given by Eq. (S4.11).

B Entropy of the simple reciprocal configuration model

To derive the entropy of the reciprocal configuration model for simple graphs, we follow the approach developed in [4]. Compared to the directed configuration model, the reciprocal version has the mutual degree sequence, i.e., the number of mutual stubs (corresponding to reciprocal edges) per node, as an additional set of parameters. We let $\mathbf{u}$ denote a vector of size N filled with ones, and we define the microcanonical partition function as a sum over a product of Dirac delta functions which define the constrained parameter values of the model,

$$\Omega(\boldsymbol{\kappa}^m, \boldsymbol{\kappa}^+, \boldsymbol{\kappa}^-) = \sum_{\mathbf{A}} \delta\left(\boldsymbol{\kappa}^m - v_D\left(\mathbf{A}\mathbf{A}^T\right)\right) \delta\left(\boldsymbol{\kappa}^+ - \mathbf{A}\mathbf{u} + v_D\left(\mathbf{A}\mathbf{A}^T\right)\right) \delta\left(\boldsymbol{\kappa}^- - \mathbf{A}^T\mathbf{u} + v_D\left(\mathbf{A}\mathbf{A}^T\right)\right), \tag{S4.25}$$

where v_D is a function that maps the diagonal elements of a $N \times N$ matrix to a N-dimensional vector. The Dirac delta functions can be expanded in terms of Fourier integrals to obtain

$$\Omega(\boldsymbol{\kappa}^m, \boldsymbol{\kappa}^+, \boldsymbol{\kappa}^-) = \int \frac{\mathrm{d}\boldsymbol{\lambda}^+ \mathrm{d}\boldsymbol{\lambda}^- \mathrm{d}\boldsymbol{\mu}}{(2\pi)^{3N}} \, e^{\boldsymbol{\lambda}^{+T}\boldsymbol{\kappa}^+ + \boldsymbol{\lambda}^{-T}\boldsymbol{\kappa}^- + \boldsymbol{\mu}^T\boldsymbol{\kappa}^m} \sum_{\mathbf{A}} e^{-\boldsymbol{\lambda}^{+T}\mathbf{A}\mathbf{u} - \boldsymbol{\lambda}^{-T}\mathbf{A}^T\mathbf{u} - \boldsymbol{\mu}^T \mathrm{diag}\left(\mathbf{A}\mathbf{A}^T\right)}, \tag{S4.26}$$

where $\boldsymbol{\lambda}^+, \boldsymbol{\lambda}^-, \boldsymbol{\mu}$ are identified as vectors of Lagrange multipliers. Compared to the classical configuration model, symmetric pairs of elements of the adjacency matrix are not independent and need to be considered simultaneously, such that

$$\begin{aligned} \sum_{\mathbf{A}} e^{-\boldsymbol{\lambda}^{+T}\mathbf{A}\mathbf{u} - \boldsymbol{\lambda}^{-T}\mathbf{A}^T\mathbf{u} - \boldsymbol{\mu}^T \mathrm{diag}\left(\mathbf{A}\mathbf{A}^T\right)} &= \sum_{\{A_{ij},A_{ji}\}=\{\{0,0\},\{1,1\},\{0,1\},\{1,0\}\}} e^{-\sum_{ij}(\mu_i - \lambda_i^+ - \lambda_i^-)A_{ij}A_{ji}} e^{-\sum_{ij}(\lambda_i^+ + \lambda_j^-)A_{ij}} \\ &= \prod_{i<j} \left(1 + e^{-\mu_i - \mu_j} + e^{-\lambda_i^+ - \lambda_j^-} + e^{-\lambda_i^- - \lambda_j^+}\right). \end{aligned} \tag{S4.27}$$

For the sake of readability, we set $s_{ij} \equiv e^{-\mu_i - \mu_j} + e^{-\lambda_i^+ - \lambda_j^-} + e^{-\lambda_j^+ - \lambda_i^-}$. To estimate $\Omega(\boldsymbol{\kappa}^m, \boldsymbol{\kappa}^+, \boldsymbol{\kappa}^-)$, we apply a Laplace approximation to its Fourier integral form, and we thus we seek to maximize the following quantity

$$NQ(\boldsymbol{\mu}, \boldsymbol{\lambda}^+, \boldsymbol{\lambda}^- | \boldsymbol{\kappa}^m, \boldsymbol{\kappa}^+, \boldsymbol{\kappa}^-) = \boldsymbol{\mu}^T\boldsymbol{\kappa}^m + \boldsymbol{\lambda}^{+T}\boldsymbol{\kappa}^+ + \boldsymbol{\lambda}^{-T}\boldsymbol{\kappa}^- + \sum_{i<j} \ln(1 + s_{ij}). \tag{S4.28}$$

The saddle point equations to be solved are thus

$$\frac{\partial Q}{\partial \mu_i} = 0 \Leftrightarrow \kappa_i^m = e^{-\mu_i} \sum_{j\neq i} \frac{e^{-\mu_j}}{1+s_{ij}}, \tag{S4.29}$$

$$\frac{\partial Q}{\partial \lambda_i^+} = 0 \Leftrightarrow \kappa_i^+ = e^{-\lambda_i^+} \sum_{j\neq i} \frac{e^{-\lambda_j^-}}{1+s_{ij}}, \tag{S4.30}$$

$$\frac{\partial Q}{\partial \lambda_i^-} = 0 \Leftrightarrow \kappa_i^- = e^{-\lambda_i^-} \sum_{j\neq i} \frac{e^{-\lambda_j^+}}{1+s_{ij}}. \tag{S4.31}$$

Here, we are only interested in the sparse graph approximation. The computation of the Hessian would be compulsory in a rigorous calculation. However, when actually evaluated, it only leads to sums of logarithmic terms in the degree sequences elements, which are negligible compared to the sums of log factorial terms in the degree sequence elements (when the graph is of finite size). This is the same observation that is found in [4], while never explicitly justified there. For our results to be consistent with the standard form of other simple network entropy expressions, we also choose to neglect the contribution of the Hessian to the sparse graph approximation of the microcanonical partition function. At large N, it is reasonable to assume $s_{ij} = o(1)$ such that the right hand terms of the saddle point equations are finite. This leads to the simplified equations

$$\kappa_i^m = K_m e^{-\mu_i}, \qquad K_m = \sum_j e^{-\mu_j}, \tag{S4.32}$$

$$\kappa_i^+ = K_- e^{-\lambda_i^+}, \qquad K_- = \sum_j e^{-\lambda_j^-}, \tag{S4.33}$$

$$\kappa_i^- = K_+ e^{-\lambda_i^-}, \qquad K_+ = \sum_j e^{-\lambda_j^+}. \tag{S4.34}$$

The constants K_m, K_+, K_- are determined by global structural constraints, which are the number of asymmetrically connected (or directed) pairs of nodes and the number of mutual edges:

$$2E_m = \sum_i \kappa_i^m = K_m^2 \Leftrightarrow K_m = \sqrt{2E_m}, \tag{S4.35}$$

$$E_d = \sum_i \kappa_i^+ = K_+ K_- \Leftrightarrow K_+ = K_- = \sqrt{E_d}. \tag{S4.36}$$

All is now set for a second-order estimation of the microcanonical partition function in s_{ij}. We have

$$\boldsymbol{\mu}^T \boldsymbol{\kappa}^m = -\sum_i \kappa_i^m \ln \kappa_i^m + E_m \ln(2E_m) \approx \ln \frac{(2E_m)!!}{\prod_i \kappa_i^m!} - E_m, \tag{S4.37}$$

$$\boldsymbol{\lambda}^{+T} \boldsymbol{\kappa}^+ + \boldsymbol{\lambda}^{-T} \boldsymbol{\kappa}^- = -\sum_i \kappa_i^+ \ln \kappa_i^+ - \sum_i \kappa_i^- \ln \kappa_i^- + E_d \ln E_d \approx \ln \frac{E_d!}{\prod_i \kappa_i^+! \kappa_i^-!} - E_d, \tag{S4.38}$$

$$s_{ij} = \frac{\kappa_i^m \kappa_j^m}{2E_m} + \frac{\kappa_i^+ \kappa_j^- + \kappa_i^- \kappa_j^+}{E_d}, \tag{S4.39}$$

and

$$\frac{1}{2}\sum_{ij}\left(s_{ij} - \frac{s_{ij}^2}{2}\right) = E_m + E_d - \frac{1}{2}\underbrace{\left(\frac{1}{2}\frac{\langle \kappa_i^{m2}\rangle^2}{\langle \kappa_i^m \rangle^2} + \frac{\langle \kappa_i^{+2}\rangle \langle \kappa_i^{-2}\rangle}{\langle \kappa_i^+\rangle \langle \kappa_i^- \rangle} + \frac{\langle \kappa_i^+ \kappa_i^- \rangle^2}{\langle \kappa_i^+\rangle \langle \kappa_i^- \rangle} + \frac{\langle \kappa_i^m \kappa_i^+\rangle \langle \kappa_i^m \kappa_i^- \rangle}{\langle \kappa_i^m \rangle \langle \kappa_i^+ \rangle}\right)}_{\Psi(\boldsymbol{\kappa}^m, \boldsymbol{\kappa}^+, \boldsymbol{\kappa}^-)}. \tag{S4.40}$$

Putting it all together we obtain

$$L_{(\boldsymbol{\kappa}^m, \boldsymbol{\kappa}^+, \boldsymbol{\kappa}^-)}(G) = \log \frac{(2E_m)!!}{\prod_i \kappa_i^m!} + \log \frac{E_d!}{\prod_i \kappa_i^+! \kappa_i^-!} - \frac{1}{2\ln 2}\Psi(\boldsymbol{\kappa}^m, \boldsymbol{\kappa}^+, \boldsymbol{\kappa}^-). \tag{S4.41}$$

Similar to the sparse approximation of the entropy of the simple graph configuration model [4], we see that the entropy for the simple graph reciprocal configuration model amounts to the multigraph codelength from which is subtracted a functional cut-off that depends on the statistics of the degree sequences.

C Subgraph contraction costs

We give in this note closed-form expressions for the putative difference in codelength which would be obtained by contracting a given subgraph in the reduced graph $H_t = (\mathcal{N}_t, \mathcal{E}_t)$. These are the expressions we use in practice in our greedy algorithm to select the most compressing subgraph at each iteration. The codelength difference for a subgraph contraction depends on the base model used to encode H_t, so we give below expressions for each of the four base models. We will for notational convenience drop the subscripts pertaining to the iteration t and thus simply refer to $H = (\mathcal{N}, \mathcal{E})$ in the remainder of this section.

C.1 Common quantities

In all steps, base models share a cost difference related to the transformation of a group of entries in the adjacency matrix $\mathbf{A} = \mathbf{A}(H)$ from $[A_{ij}]$ to $[A'_{ij}]$ caused by the contraction of a given subgraph $s = (\nu, \epsilon) \in H$. This difference is given by

$$\ell_{\mathbf{A}}(s) \; = \sum_{i\in\nu} \log\left[\frac{k_i^+(s)!}{\prod_{j\in\partial s} A_{ij}!} \times \frac{k_i^-(s)!}{\prod_{j\in\partial s} A_{ji}!}\right], \tag{S4.42}$$

where the subgraph's neighborhood ∂s is the set of nodes in H that are connected to a node of s, and

$$k_i^+(s) \; = \sum_{j\in\partial s} A_{ij}, \tag{S4.43}$$

$$k_i^-(s) \; = \sum_{j\in\partial s} A_{ji}. \tag{S4.44}$$

$$\tag{S4.45}$$

In the following, we will denote by $n = |\nu|$ the subgraph size and by $e = |\epsilon|$ the number of edges s holds. We furthermore denote by $e_d = \frac{1}{2}\sum_{i,j\in\nu} |A_{ij} - A_{ji}|$ the number of directed (asymmetric) edges and by $e_m = \frac{1}{2}(e - e_d)$ the number of mutual edges of s. The number of nodes in H is denoted N, the number of edges E, the number of mutual edges E_m, and the number of (asymmetric) directed edges E_d.

C.2 Base model complexity cost

In the inference of our model, the entropy of the base model, S_ϕ, is not the only term that is affected when contracting a subgraph. The base model parameters $\phi(H)$ may also change, which, in turn, may change the codelength needed to encode them, and must thus also be taken into account in the putative codelength difference.

C.2.1 Positive integer

We let a denote a positive integer. It could represent a number of edges or nodes, the maximum degree, etc. As was described in the section "Compression, model selection, and hypothesis testing", a can be encoded using $L_{\mathbb{N}}(a)$ bits. Thus, if the contraction of a subgraph s induces the variation $a \longrightarrow a + \Delta a(s)$, then the associated compression (codelength difference) is

$$\Delta L_{\mathbb{N}}(a, s) = L_{\mathbb{N}}(a) - L_{\mathbb{N}}(a + \Delta a(s)) = \log \frac{a(a+1)}{[a + \Delta a(s)][a + \Delta a(s) + 1]}. \tag{S4.46}$$

For instance, for the ER model, a subgraph contraction changes the base model's parameters as $\Delta E(s) = -e$ and $\Delta N(s) = 1 - n$, leading to a change in codelength of

$$\Delta L_{\mathbb{N}}(E, s) = \log \frac{E(E+1)}{[E-e][E-e+1]} \tag{S4.47}$$

for describing E, and of

$$\Delta L_{\mathbb{N}}(N, s) = \log \frac{N(N+1)}{[N-n+1][N-n+2]} \tag{S4.48}$$

for describing N.

C.2.2 Sequences of positive integers

Let $\mathbf{a} = (a_i)$ be a sequence of N positive integers. For our purpose, $\mathbf{a}$ represents a sequence of node degrees (i.e., the in,- out-, or mutual degrees of H). The sequence $\mathbf{a}$ is described either by a uniform code or a plug-in code (Eq. (S4.11)) that both depend on the range of values distributed in $\mathbf{a}$. We let the maximum value of the sequence be denoted $Q \equiv \max \mathbf{a}$ (Δ in the main text), and the mininum value $q \equiv \min \mathbf{a}$ (δ in the main text). For each distinct value μ in $\mathbf{a}$, we let $r_\mu \equiv \sum_{i=1}^{N} \delta(a_i, \mu)$ denote its frequency. Let $a(s) \equiv a_{i_s}$ be the new sequence element added to $\mathbf{a}$, after s is contracted into a supernode, which is labeled i_s. It is given by

$$a(s) = \sum_{i \in \nu} a_i - a_0(s), \tag{S4.49}$$

where $a_0(s)$ is related to the deleted internal edges of s or the concatenation of subgraph neighborhoods into multiedges. Let us review how supernode degrees are computed in the configuration model and its reciprocal conterpart to show that the above expression holds for the present study.

In the case of the configuration model, the evaluation of the out-degree of a new supernode is

$$k^+(s) = \sum_{i \in \nu} \sum_{j \in \partial s} A_{ij} \tag{S4.50}$$

$$= \sum_{i \in \nu} \left(\sum_{j} A_{ij} - \sum_{j \in s} A_{ij} \right) \tag{S4.51}$$

$$= \sum_{i \in \nu} k_i^+ - e, \tag{S4.52}$$

such that one identifies $k_0(s) = e$. For the in-degree sequence, the expression holds by the change of notation $+ \longrightarrow -$.

For the reciprocal configuration model, three degree sequences are involved. Starting with the directed out-degree,

$$\kappa^+(s) = \sum_{j \in \partial s} \max \left(\sum_{i \in \nu} (A_{ij} - A_{ji}), 0 \right) \tag{S4.53}$$

$$= \frac{1}{2} \sum_{i \in \nu} \sum_{j \in \partial s} (A_{ij} - A_{ji}) + \frac{1}{2} \sum_{j \in \partial s} \left| \sum_{i \in \nu} (A_{ij} - A_{ji}) \right| \tag{S4.54}$$

$$= \sum_{i \in \nu} \kappa_i^+ - \kappa_0(s), \tag{S4.55}$$

with

$$\kappa_0(s) = e_d + \frac{1}{2} \sum_{j \in \partial s} \left(\sum_{i \in \nu} |A_{ij} - A_{ji}| - \left| \sum_{i \in \nu} (A_{ij} - A_{ji}) \right| \right). \tag{S4.56}$$

The directed in-degree is identical after the change of notation $+ \longrightarrow -$. The mutual degree of a supernode is simply given by its relationship to the configuration model's in- (or out-) degree and to the mutual degree,

$$\kappa^m(s) = k^+(s) - \kappa^+(s) = k^-(s) - \kappa^-(s) \tag{S4.57}$$

$$= \sum_{i \in s} (k_i^+ - \kappa_i^+) - [k_0(s) - \kappa_0(s)] \tag{S4.58}$$

$$= \sum_{i \in s} \kappa_i^m - \kappa_0^m(s) \tag{S4.59}$$

The supernode degree and node degrees of the subgraph's nodes are not the only sequence elements modified by the subgraph contraction. Properties of the subgraph's neighborhood are also likely to evolve if degree sequences are correlated, which is the case of the reciprocal configuration model. After the contraction of s, we will denote by $\Delta a_j(s)$, the variation of the element $a_j \longrightarrow a_j + \Delta a_j(s)$, associated to a network property of node $j \in \partial s$. Variations of the directed in- and out-degrees are

equal:

$$\begin{aligned}\Delta\kappa_j^+(s) &= \max\left(\sum_{i\in\nu}(A_{ji}-A_{ij}),0\right) - \sum_{i\in\nu}\max(A_{ji}-A_{ij},0)\\ &= \frac{1}{2}\left|\sum_{i\in\nu}(A_{ji}-A_{ij})\right| - \frac{1}{2}\sum_{i\in\nu}|A_{ji}-A_{ij}|\\ &= \Delta\kappa_j^-(s) \equiv \Delta\kappa_j(s) \end{aligned} \tag{S4.60}$$

The case of the mutual degree is deduced by the conservation of the in- and out-degree of the subgraph's neighbors:

$$\Delta k_j^+(s) = \Delta k_j^-(s) = 0 \tag{S4.61}$$

$$\Leftrightarrow \Delta\kappa_j^m(s) = -\Delta\kappa_j(s) \tag{S4.62}$$

One can write how the distribution $\{r_\mu\}$ transforms into $\{r_\mu + \Delta r_\mu(s)\}$.

$$\Delta r_\mu(s) = \delta(a(s),\mu) - \sum_{i\in s}\delta(a_i,\mu) + \sum_{j\in\partial s}[\delta(a_j+\Delta a_j(s),\mu) - \delta(a_j,\mu)] \tag{S4.63}$$

Updates of $Q \longrightarrow Q + \Delta Q(s)$ and $q \longrightarrow q + \Delta q(s)$ are naturally determined by the evolution of the sequence $\mathbf{a} \longrightarrow \mathbf{a} + \Delta\mathbf{a}(s)$, and by how the distribution $\{r_\mu\}$ is shifted, due to the contraction of s, by the incremental distribution $\{\Delta r_\mu(s)\}$. Consider first the case of the update of the maximum. A first scenario could be an augmentation of the maximum, $\Delta Q(s) > 0$, by either the new supernode or an increase of a subgraph's neighbor degree, i.e., $\Delta a_j(s) > 0$. Let $Q'(s)$ be the maximum between a supernode degree and one of the updated subgraph's neighbors degrees:

$$Q'(s) = \max\left(a(s), \max_{j\in\partial s}\{a_j + \Delta a_j(s)\}\right) \tag{S4.64}$$

A second scenario, when $Q'(s) < Q$, is the possible extinction of the maximum, i.e., $\Delta r_Q(s) = -r_Q$. Introducing the quantity $\Delta Q'(s) = \max(Q'(s) - Q, 0)$, the difference in maxima is expressed as

$$\Delta Q(s) = \Delta Q'(s) + \delta(\Delta Q'(s),0)\delta(\Delta r_Q(s),-r_Q)\sum_{\mu=0}^{Q-1}(\mu - Q)\mathfrak{g}_\mu^Q(s) \tag{S4.65}$$

where $\mathfrak{g}_\mu^Q(s)$ in an indicator function that returns 1 when μ is the highest value below Q in $\mathbf{a}$ after the update, and 0 otherwise. It can be formally written as:

$$\mathfrak{g}_\mu^Q(s) = [1 - \delta(\Delta r_\mu(s), -r_\mu)]\prod_{\mu'=\mu+1}^{Q-1}\delta(\Delta r_{\mu'}(s), -r_{\mu'}) \tag{S4.66}$$

The first term on the RHS of Eq. S4.65 corresponds to the case where the maximum of the sequence is increased by the insertion of a supernode or the restructuring of the subgraph's neighborhood. The second term is the alternative scenario where the maximum is decreased and must be searched within $\mathbf{a}$. The variation in minima $\Delta q(s)$ is naturally similar to Eq. S4.65. Let $q'(s)$ be the minimum between a supernode degree and one of the updated subgraph's neighbor degrees:

$$q'(s) = \min\left(a(s), \min_{j\in\partial s}\{a_j + \Delta a_j(s)\}\right) \tag{S4.67}$$

Introducing $\Delta q'(s) = -\min(q - q'(s), 0)$, the difference in minima is expressed as

$$\Delta q(s) = \Delta q'(s) + \delta(\Delta q'(s),0)\delta(\Delta r_q(s),-r_q)\sum_{\mu=q+1}^{Q+\Delta Q(s)}(\mu - q)\mathfrak{g}_\mu^q(s) \tag{S4.68}$$

where $\mathfrak{g}_\mu^q(s)$ in an indicator function that returns 1 when μ is the lowest value greater than q in $\mathbf{a}$ after the update, and 0 otherwise. It can be formally written as:

$$\mathfrak{g}_\mu^q(s) = [1 - \delta(\Delta r_\mu(s), -r_\mu)]\prod_{\mu'=q+2}^{\mu}\delta(\Delta r_{\mu'}(s), -r_{\mu'}) \tag{S4.69}$$

The first term on the RHS of Eq. S4.68 corresponds to the case where the minimum of the sequence is decreased by the insertion of a supernode or the restructuring of the subgraph's neighborhood. The second term is the alternative scenario where the minimum is increased and must be searched within $\mathbf{a}$. All necessary quantities involved in putative codelength differences of integer sequences have been determined. Let us now give their exact expressions.

Uniform code. A uniform encoding of $\mathbf{a}$ corresponds to N products of a uniform probability distribution over q to Q,

$$L_U(\mathbf{a}) = N \log(Q - q + 1) + L_{\mathbb{N}}(Q) + L_{\mathbb{N}}(q). \tag{S4.70}$$

The codelength difference is

$$\begin{aligned}\Delta L_U(\mathbf{a}, s) = &-(N - n + 1) \log\left(1 + \frac{\Delta Q(s) - \Delta q(s)}{Q - q + 1}\right) + (n - 1) \log(Q - q + 1) \\ &+ \Delta L_{\mathbb{N}}(Q, s) + \Delta L_{\mathbb{N}}(q, s).\end{aligned} \tag{S4.71}$$

Plug-in code. The plug-in code is a function of $\{r_\mu\}$ and a hyperparameter λ, that constrains the shape of the prior. Two different values for λ were considered in the main text, $\lambda = 1/2$ (Jeffreys prior) and $\lambda = 1$ (uniform prior). The plug-in code of a sequence is characterized by three entities: N, $\{r_\mu\}_{q\leq\mu\leq Q}$, and $\Lambda \equiv \Lambda(Q, q) = (Q - q + 1)\lambda$:

$$L_\lambda(\mathbf{a}) = \log \frac{\Gamma(N + \Lambda)}{\Gamma(\Lambda)} + (Q - q + 1) \log \Gamma(\lambda) - \sum_{q\leq\mu\leq Q} \log \Gamma(r_\mu + \lambda). \tag{S4.72}$$

Let $\Delta\Lambda(Q, q, s)$ the variation following the contraction of s, $\Lambda(Q, q) \longrightarrow \Lambda(Q, q) + \Delta\Lambda(Q, q, s)$. The latter is determined by how the maximum and minimum of Q and q are closer or more distant after the subgraph contraction. One can independently treat the case where Q or q changes. Thus, we adopt the following decomposition,

$$\Delta\Lambda(Q, q, s) = \Delta\Lambda(Q, s) + \Delta\Lambda(q, s) \tag{S4.73}$$

$$= [\Delta Q(s) - \Delta q(s)]\lambda \tag{S4.74}$$

The update of the plug-in code after a subgraph contraction is divided into multiple cases, depending on how the contraction of a subgraph s affects N, $\{r_\mu\}_{q\leq\mu\leq Q}$, and $\Lambda(Q, q)$. All in all, the plug-in codelength difference is

$$\begin{aligned}\Delta L_\lambda(\mathbf{a}, s) = &\log \frac{\Gamma(N + \Lambda)}{\Gamma(N - n + 1 + \Lambda + \Delta\Lambda(Q, q, s))} + \log \frac{\Gamma(\Lambda + \Delta\Lambda(Q, q, s))}{\Gamma(\Lambda)} \\ &+ \sum_{\mu=\mu_{\min}}^{\mu_{\max}} \log \frac{\Gamma(r_\mu + \Delta r_\mu(s) + \lambda)}{\Gamma(r_\mu + \lambda)} + \Delta L_{\mathbb{N}}(N, s) + \Delta L_{\mathbb{N}}(Q, s) + \Delta L_{\mathbb{N}}(q, s),\end{aligned} \tag{S4.75}$$

where $\mu_{\min} = \min(q, q + \Delta q(s))$ and $\mu_{\max} = \max(Q, Q + \Delta Q(s))$.

C.3 Erdős-Rényi model

For the ER model, (N, E) will change to $(N - n + 1, E - e)$ after the contraction of s. The putative codelength difference is then given by

$$\begin{aligned}\Delta L_{(N,E)}(H, s) = &\, e \log[(N - n)(N - n + 1)] + E \log \frac{N(N - 1)}{(N - n)(N - n + 1)} \\ &- \log \frac{E!}{(E - e)!} - \ell_{\mathbf{A}}(s).\end{aligned} \tag{S4.76}$$

C.4 Reciprocal Erdős-Rényi model

For the reciprocal ER model, the variation of the number of mutual edges and directed edges do not only depend on e_d and e_m because the formation of multiedges (as stacked single edges) changes

the number of mutual edges E_m and the number of directed edges E_d in H. The variations of the number of mutual edges $\Delta E_m(s)$ and of the number of directed edges $\Delta E_d(s)$ are given by

$$\Delta E_m(s) = -e_m + \sum_{j\in\partial s}\left[\min\left(\sum_{i\in\nu}A_{ij}, \sum_{i\in\nu}A_{ji}\right) - \sum_{i\in\nu}\min(A_{ij}, A_{ji})\right] \tag{S4.77}$$

$$= -e_m + \frac{1}{2}\sum_{j\in\partial s}\left(\sum_{i\in\nu}|A_{ij} - A_{ji}| - \left|\sum_{i\in\nu}(A_{ij} - A_{ji})\right|\right), \tag{S4.78}$$

and

$$\Delta E_d(s) = -\, e - 2\Delta E_m(s) \tag{S4.79}$$

$$= -\, e_d - \sum_{j\in\partial s}\left(\sum_{i\in\nu}|A_{ij} - A_{ji}| - \left|\sum_{i\in\nu}(A_{ij} - A_{ji})\right|\right). \tag{S4.80}$$

The codelength has two part, one for the directed edges and another for the mutual edges. For the directed edges, one can adapt Eq. S4.76, and replace e by $\Delta E_d(s)$:

$$\Delta L_{(N,E_d)}(H^{\text{asym}}, s) = -\Delta E_d(s)\log\left[(N-n)(N-n+1)\right] + E_d \log\frac{N(N-1)}{(N-n)(N-n+1)} - \log\frac{E_d!}{(E_d + \Delta E_d(s))!} - \ell_{\mathbf{A}^{\text{asym}}}(s). \tag{S4.81}$$

For the mutual part, the putative codelength difference is:

$$\Delta L_{(N,E_m)}(H^{\text{sym}}, s) = -\Delta E_m(s)\log\left[\frac{(N-n)(N-n+1)}{2}\right] + E_m \log\frac{N(N-1)}{(N-n)(N-n+1)} - \log\frac{E_m!}{(E_m + \Delta E_m(s))!} - \ell_{\mathbf{A}^{\text{sym}}}(s). \tag{S4.82}$$

where $\ell_{\mathbf{A}^{\text{sym}}}(s)$ is the undirected version of Eq. S4.42, where only one of two terms inside the log needs to be kept. Finally, the codelength difference can be written as

$$\Delta L_{(N,E_m,E_d)}(H, s) = \Delta L_{(N,E_d)}(H^{\text{asym}}, s) + \Delta L_{(N,E_m)}(H^{\text{sym}}, s) \tag{S4.83}$$

C.5 Configuration model

The codelength difference when contracting a subgraph s for the configuration model is

$$\Delta L_{(\mathbf{k}^+,\mathbf{k}^-)}(H, s) = \log\frac{E!}{(E-e)!} + \log\left[\frac{k^+(s)!}{\prod_{i\in\nu}k_i^+!} \times \frac{k^-(s)!}{\prod_{i\in\nu}k_i^-!}\right] - \ell_{\mathbf{A}}(s), \tag{S4.84}$$

where $k_i^+ = k_i^+(H)$, and $k_i^- = k_i^-(H)$ as above, and

$$k^\pm(s) \ = \sum_{i\in\nu}k_i^\pm(s) = \sum_{i\in\nu}k_i^\pm - e. \tag{S4.85}$$

C.6 Reciprocal configuration model

Finally, the codelength difference for the reciprocal configuration model is equal to

$$\Delta L_{(\boldsymbol{\kappa}^m,\boldsymbol{\kappa}^+,\boldsymbol{\kappa}^-)}(H, s) = \log\frac{E_d!}{[E_d + \Delta E_d]!} + \log\frac{(2E_m - 1)!!}{[2(E_m + \Delta E_m) - 1]!!} + \log\left[\frac{\kappa^+(s)!}{\prod_{i\in\nu}\kappa_i^+!} \times \frac{\kappa^-(s)!}{\prod_{i\in\nu}\kappa_i^-!} \times \frac{\kappa^m(s)!}{\prod_{i\in\nu}\kappa_i^m!}\right] + \sum_{j\in\partial s}\log\left[\frac{(\kappa_j^+ + \Delta\kappa_j(s))!}{\kappa_j^+!} \times \frac{(\kappa_j^- + \Delta\kappa_j(s))!}{\kappa_j^-!} \times \frac{(\kappa_j^m - \Delta\kappa_j(s))!}{\kappa_j^m!}\right] - \ell_{\mathbf{A}^{\text{asym}}}(s) - \ell_{\mathbf{A}^{\text{sym}}}(s). \tag{S4.86}$$

where

$$\kappa^{\pm}(s) = \sum_{i\in\nu} \kappa_i^{\pm} + \frac{1}{2}\Delta E_d(s) - \frac{e_d}{2} \tag{S4.87}$$

$$\kappa^{m}(s) = \sum_{i\in\nu} \kappa_i^{m} + \Delta E_m(s) \tag{S4.88}$$

are respectively the directed and mutual degrees of the future supernode. The $\{\Delta\kappa_j(s)\}_{j\in\partial s}$ are respectively variations of the directed degrees of the subgraph neighborhood due to its contraction (see Eq. S4.60 for their expressions).

C.7 Motif-based code

Based on the previous subsections, we can give the complete putative codelength difference when contracting a subgraph. As a reminder, the codelength of our model is

$$L(G,\theta) = L(\Gamma,\mathcal{S}) + L(H,\phi) + L(\mathcal{V}|H,\mathcal{S}) + L(G|H,\mathcal{V},\mathcal{S},\Gamma), \tag{S4.89}$$

where $\theta = \{H,\phi,\mathcal{S},\mathcal{V},\Gamma\}$. H is the reduced multigraph, Γ is the set of all discovered graphlets, $\mathcal{S}$ is the graphlet multiset (a proxy for a set of subgraphs), $\mathcal{V}$ are H's node labels identifying supernodes, and ϕ are the parameters of the dyadic base model. Let us give the subgraph-contraction-induced cost for all terms of the above equation.

The update of the encoding cost of the graphlet set and multiset, $L(\Gamma,\mathcal{S})$ (see Eq. (6) in the "Methods" section), is seen as an extension of $\mathcal{S}$ by α, the label of the graphlet to which s is isomorphic. We choose to encode $\mathcal{S}$ as an ordered multiset of elements, that are independently sampled from Γ and their respective frequency in $\mathcal{S}$ is encoded by a uniform distribution over the range one to $m_{\max}$. The minimum value of $m_{\max}$ is one. Two exclusive scenarios may occur for a non-zero update cost. Either an occurrence of the most represented graphlet in $\mathcal{S}$ is again selected and leads to an incremental increase of $m_{\max}$, or s is isomorphic to a different $\alpha \notin \mathcal{S}$. Denoting by $\mathcal{A}$ the unique set of elements of $\mathcal{S}$,

$$\begin{aligned}\Delta L(\Gamma,\mathcal{S},s) = -\sum_{\alpha\in\mathcal{A}} \mathbb{I}(s\cong g_\alpha)\Big\{&\delta(m_\alpha,m_{\max})\left[|\mathcal{A}|\log\left(1+\frac{1}{m_{\max}}\right)+\log\left(1+\frac{2}{m_{\max}}\right)\right] \\ &+\delta(m_\alpha,0)\left(\log|\Gamma|+\log m_{\max}\right)\Big\}\end{aligned} \tag{S4.90}$$

where $\mathbb{I}(s\cong g_\alpha)$ is an indicator function that is one if s is isomorphic to the graphlet of canonical label α.

The update of the encoding of the supernode labels, $L(\mathcal{V}|H,\mathcal{S})$ (see Eq. (7) in the "Methods" section), is, again, affected by the graph size, the growth of the supernode number and the incremental increase of a graphlet occurrence. Denoting by $M \equiv \sum_{\alpha'} m_{\alpha'} = |\mathcal{S}|$ the number of supernodes,

$$\Delta L(\mathcal{V},s|H,\mathcal{S}) = \log\binom{N}{M} - \log\binom{N-n+1}{M+1} + \sum_{\alpha\in\mathcal{A}}\mathbb{I}(s\cong g_\alpha)\log\frac{m_\alpha+1}{M+1} \tag{S4.91}$$

The update of reconstruction cost from H to G depends on the reduced graph size, the associated graphlet orientation number and the subgraph's neighborhood:

$$\begin{aligned}\Delta L(G,s|H,\mathcal{V},\mathcal{S},\Gamma) = \log\frac{(N-n+1)!}{N!} &+ \sum_{\alpha\in\mathcal{A}}\mathbb{I}(s\cong g_\alpha)\frac{n_\alpha!}{|\mathrm{Aut}(\alpha)|} \\ &+ \sum_{j\in\mathcal{N}(H)\setminus\mathcal{V}}\log\binom{n}{\sum_{i\in\nu}A_{ij}}\binom{n}{\sum_{i\in\nu}A_{ji}} + \sum_{j_{s'}\in\mathcal{V}}\log\binom{nn_{j_s'}}{\sum_{i\in\nu}A_{ij_{s'}}}\binom{nn_{j_s'}}{\sum_{i\in\nu}A_{j_{s'}i}},\end{aligned} \tag{S4.92}$$

where $n_{j_s'}$ is the subgraph size relative to the supernode $j_{s'}\in\mathcal{V}$, replacing the subgraph s'. The two sums represent the encoding of the nodes' neighbors within s, i.e., how to distribute the multiedge among the nodes that would be deleted. The first sum corresponds to regular node neighbors, while the second sum corresponds to supernode neighbors.

Finally, $\Delta L_\phi(H,s)$ being given by Eqs. (S4.76),(S4.83),(S4.84),(S4.86) and $\Delta L(\phi,s)$ by Eqs. (S4.46),(S4.71),(S4.75) the complete putative codelength difference is:

$$\Delta L(G,\theta,s) = \Delta L_\phi(H,s) + \Delta L(\phi,s) + \Delta L(G,s|H,\mathcal{V},\mathcal{S},\Gamma) + \Delta L(\mathcal{V},s|H,\mathcal{S}) + \Delta L(\Gamma,\mathcal{S},s) \tag{S4.93}$$

References

1. Peixoto TP. Nonparametric Bayesian inference of the microcanonical stochastic block model. Phys Rev E. 2017;95(1):012317. doi:10.1103/PhysRevE.95.012317.

2. Grünwald P, Roos T. Minimum description length revisited. International Journal of Mathematics for Industry. 2020;doi:10.1142/S2661335219300018.

3. Fosdick BK, Larremore DB, Nishimura J, Ugander J. Configuring Random Graph Models with Fixed Degree Sequences. SIAM Rev. 2018;60(2):315–355. doi:10.1137/16M1087175.

4. Bianconi G. Entropy of network ensembles. Phys Rev E. 2009;79(3):036114. doi:10.1103/PhysRevE.79.036114.

5. Grünwald PD. The Minimum Description Length Principle. Penguin Book; 2007.

6. Bloem P, de Rooij S. Large-scale network motif analysis using compression. Data Min Knowl Disc. 2020;34(5):1421–1453. doi:10.1007/s10618-020-00691-y.

7. Jovanic T, Schneider-Mizell CM, Shao M, Masson JB, Denisov G, Fetter RD, et al. Competitive Disinhibition Mediates Behavioral Choice and Sequences in Drosophila. Cell. 2016;167(3):858–870.e19. doi:10.1016/j.cell.2016.09.009.

8. Winding M, Pedigo BD, Barnes CL, Patsolic HG, Park Y, Kazimiers T, et al. The connectome of an insect brain. Science. 2023;379(6636):eadd9330.

9. Gilbert CD, Li W. Top-down influences on visual processing. Nature Reviews Neuroscience. 2013;14(5):350–363.

10. Bahl A, Engert F. Neural circuits for evidence accumulation and decision making in larval zebrafish. Nature neuroscience. 2020;23(1):94–102.

11. Jarrell TA, Wang Y, Bloniarz AE, Brittin CA, Xu M, Thomson JN, et al. The connectome of a decision-making neural network. science. 2012;337(6093):437–444.
