# Supplementary material for: Compression-based inference of network motif sets
Source: PLoS Comput Biol. 2024 Oct 10;20(10):e1012460. doi: 10.1371/journal.pcbi.1012460 (PMC11495616; doi:10.1371/journal.pcbi.1012460)
Supplement: S5 Text — (PDF) [file pcbi.1012460.s005.pdf]

S5 Text: Generating random graphs from the null models

We generate random graphs from the four different null models by employing Markov Chain Monte Carlo (MCMC) edge switching procedures that constrain the corresponding graph features while maximally randomizing the graph structure under this constraint.

Erdős-Rényi model. To sample graphs from the Erdős-Rényi (ER) model starting from a given network G, we switch in each iteration of the edge swapping a random edge $(i, j) \in \mathcal{E}(G)$ with a random non-edge $(k, l) \in \mathcal{E}(\bar{G})$, where $\bar{G}$ is the complement graph of G, i.e., $A_{ij}(\bar{G}) = 1 - A_{ij}(G)$ [1]. The procedure conserves N and E, but otherwise generates maximally random networks.

Reciprocal Erdős-Rényi model. The procedure for generating random graphs from the reciprocal ER (RER) model is very similar to the one for the ER model, except that we additionally enforce the conservation of the numbers of mutual and single edges. This is done by explicitly distinguishing two types of edge switching, selected randomly at every step, one that switches single edges, the other that switches mutual edges. Unconnected node pairs are sampled rather than non-edges because we must ensure that a directed edge switch will not lead to the creation of new mutual edge. The procedure thus conserves N, E_d, and E_m.

Configuration model. To sample the configuration model (CM), we employ the "Maslov-Sneppen" edge-swapping algorithm algorithm to generate random graphs that share a fixed degree sequence. Let (i, j) and (k, l) be two edges of G, then the edge-swap is defined by the transformations $(i, j) \longrightarrow (i, l)$ and $(k, l) \longrightarrow (k, j)$. If the edge swap leads to a loop, i.e., $i = l$ or $k = j$, then the swap is rejected [1].

Reciprocal configuration model. The generative procedure for sampling the reciprocal CM (RCM) combines those of the CM and the RER model. As for the RER model, each step of the algorithm is either a mutual or single (directed) edge swap selected at random. The edge swap is then performed following the Maslov-Sneppen procedure as described above, either between a pair of directed or a pair of reciprocal edges. If the edge swap is directed, the reciprocal connection of the newly formed edge must be empty, otherwise, the swap is rejected [2].

References

1. Orsini C, Dankulov MM, Colomer-de Simón P, Jamakovic A, Mahadevan P, Vahdat A, et al. Quantifying randomness in real networks. Nat Commun. 2015;6(1):1–10. doi:10.1038/ncomms9627.
2. Milo R, Itzkovitz S, Kashtan N, Levitt R, Shen-Orr S, Ayzenshtat I, et al. Superfamilies of Evolved and Designed Networks. Science. 2004;303(5663):1538–1542. doi:10.1126/science.1089167.
