# Supplementary material for: Compression-based inference of network motif sets
Source: PLoS Comput Biol. 2024 Oct 10;20(10):e1012460. doi: 10.1371/journal.pcbi.1012460 (PMC11495616; doi:10.1371/journal.pcbi.1012460)
Supplement: S6 Text — Fig A in S6. Text. Distribution of graph polynomial root (GPR) values of all 3–5-node graphlets. The minimum value of the GPR, for five-node graphlets, is 1/5. It would be 0 in an infinite, maximally asymmetric graph, e.g., one where the automorphism group is a singleton. A GPR of 1, i.e., its maximum value for any graph size, represent maximally symmetric graphs, e.g., cliques. The symmetry of inferred motif sets in Fig 5 in the “Results” section should be interpreted knowing that the GPR is bounded between 0.2 and 1. (PDF) [file pcbi.1012460.s006.pdf]

S6 Text: Measures of graphlet topology

Density. The *density* ρ measures the fraction of node pairs in a simple graph $G = (N, E)$ that are connected by an edge [1],

$$\rho = \frac{E}{N(N-1)}. \tag{S4.1}$$

A simple graph is said to be *sparse* if its density is close to zero, and *dense* if its density is close to one.

Reciprocity. The *reciprocity* r measures the fraction of edges in a graph G that are reciprocated [1],

$$r = \frac{1}{E} \sum_{ij} A_{ij} A_{ji}, \tag{S4.2}$$

where $A_{ij} \in \{0, 1\}$ are the entries of the adjacency matrix for G.

Number of cycles. The *number of cycles* of a simple graph G is the number of *distinct* closed paths in G where no node appears twice, and where two cycles are distinct if one is not a cyclic permutation of the other. We calculate the number of cycles using Johnson's algorithm [**?**] implemented in the Python module NetworkX [1].

Graph polynomial root. The *graph polynomial root* (GPR) is a measure of the symmetry of a graph. It is related to the so-called orbit-polynomial [2] $\Pi_G(z)$ and allows a ranking of graphs based on the distribution of their orbit sizes. Let c_{o_l} be the number of orbits of size o_l, where $l \in \{1, 2, \ldots, L\}$ and L is the number of different orbit sizes. The graph polynomial is then defined as

$$\Pi_G(z) = \sum_{l=1}^{L} c_{o_l} z^{o_l}. \tag{S4.3}$$

The GPR, denoted z^*, is the unique solution of the following equation

$$\Pi_G(z^*) = 1, \tag{S4.4}$$

which can be solved numerically. Orbit sizes are determined using McKay's `nauty` algorithm [3]. A strong degree of symmetry is affiliated with a high GPR, while an asymmetric structure corresponds to a low GPR. Fig **A**. shows the distribution of values of the GPR of all 9 364 3- to 5-node graphlets.

References

1. Hagberg A, Conway D. Networkx: Network analysis with python. URL: https://networkx github io. 2020;.

2. Dehmer M, Chen Z, Emmert-Streib F, Mowshowitz A, Varmuza K, Feng L, et al. The orbit-polynomial: a novel measure of symmetry in networks. IEEE access. 2020;8:36100–36112.

3. McKay BD, Piperno A. Practical graph isomorphism, II. Journal of symbolic computation. 2014;60:94–112.

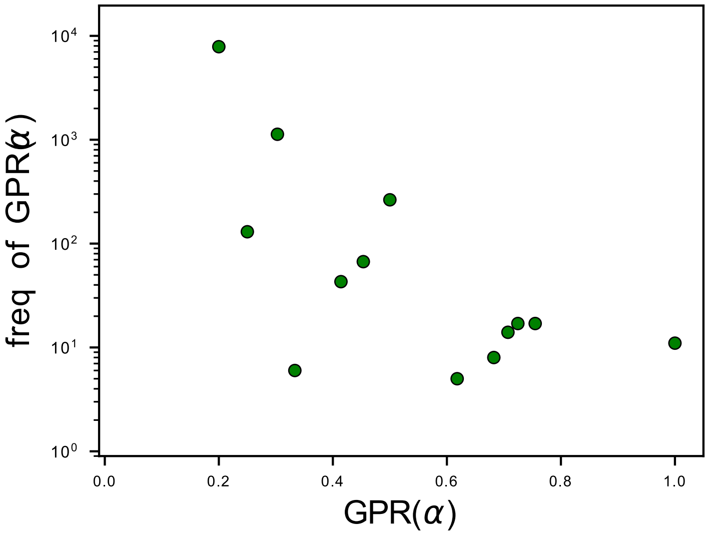

Fig A: **Distribution of graph polynomial root (GPR) values of all 3- to 5-node graphlets.** The minimum value of the GPR is 1/5 for five-node graphlets. It would be 0 in an infinite, maximally asymmetric graph, e.g., one where the automorphism group is a singleton. A GPR of 1, i.e., its maximum value for any graph size, represent maximally symmetric graphs, i.e., cliques or empty graphs. The symmetry of inferred motif sets in Fig 5 in the "Results" section should be interpreted knowing that the GPR is bounded between 0.2 and 1.
