# Supplementary material for: Compression-based inference of network motif sets
Source: PLoS Comput Biol. 2024 Oct 10;20(10):e1012460. doi: 10.1371/journal.pcbi.1012460 (PMC11495616; doi:10.1371/journal.pcbi.1012460)
Supplement: Attachment — Submitted filename: benichou.pdf [file pcbi.1012460.s015.pdf]

The authors present an inference method to identify motifs in graphs using the minimum description length principle for model selection.

I think that this is a nice contribution and is a good fit for PLoS Computation Biology.
I think that the authors do a good job of testing their approach in different scenarios, and also of considering a wide range of null models to make ‘real’ model selection. Overall, I think that this is a very robust approach to the problem of motif identification that should be adopted by the scientific community.

I have a few suggestions/comments that the authors could consider to improve their manuscript:

- Writing. Overall, I think that the manuscript is well-written but is somewhat dense. I fully understand this is a methodological paper (and I am all for it). However, for the general audience it might be a little bit too much to have to go through all the derivations to get to the plots they can understand. I would consider shortening some parts --- for instance, the expressions for the description lengths of the null models have already been derived somewhere else, so maybe these explanations are not really necessary and a table/list is enough? This is just a suggestion. I understand that the authors might want to keep consistency above all, but the paper is long.

- Algorithm and degeneracy. If I understand correctly each node in the original network can only belong to one motif which corresponds to the one motif that compresses the network the most. Are there ever ties, i.e. can a node belong to two motifs that are equally compressive? What happens then? This also leads to the question, is there a unique set of motifs that compresses the network the most, or are there several similarly compressive options (i.e. if I run the algorithm twice, do I get the same answer?). One would expect some level of degeneracy due to errors in connectome reconstruction. Then, can one really assign a set of motifs to a specific connectome without taking into account other compressive sets of motifs?

- Motif size. I imagine that there is a limitation to the size of the motifs one can consider at a computational level, but it was not clear to me what is the threshold value the authors used for that parameter. Is it possible to come up with an estimate of the largest motif that it would make sense to consider (given for instance the number of nodes in the networks and its density)? Intuitively, the larger the motif the more costly to describe, but also the most potentially compressive, so is there some size beyond which description cost outweighs compressiveness?

- Motif size and density. The authors show in Fig 3 that for 300 nodes and rho=0.1 ‘low density’ motifs are typically undetectable, and high density motifs (i.e. cliques) are detectable. Then, in Fig. 6 the authors show that the recurrent motifs across networks are, in fact, cliques. This makes me wonder, is this because of an existing ‘detectability threshold’ so that these dense motifs are the only motifs that are detectable (as Fig 5 also suggests)? This is connected to my previous question of wether there is a theoretical limit to the density of motifs that one can detect. Intuitively, cliques are cheap to describe but medium density motifs are more costly and therefor never selected. Is my intuition correct?

- Limitations. Form what the authors said, users need a super cluster to get these results. Can they comment on how this limits the applicability of their approach?

Minor edit:
If I understand correctly lines 211-212 are a retell of line 206.
