# Supplementary material for: Compression-based inference of network motif sets
Source: PLoS Comput Biol. 2024 Oct 10;20(10):e1012460. doi: 10.1371/journal.pcbi.1012460 (PMC11495616; doi:10.1371/journal.pcbi.1012460)
Supplement: Attachment — Submitted filename: Resubmission letter.pdf [file pcbi.1012460.s016.pdf]

Date: June 8, 2024
Laboratory: Decision and Bayesian Computation
Departments: Computational Biology, Neuroscience
Affiliation: CNRS UMR 3571, Inria
Institution: Institut Pasteur
Contact: Christian L. Vestergaard
Phone: +33-144389267
E-mail: christian.vestergaard@cnrs.fr; alexis.benichou@pasteur.fr
Subject: Resubmission of manuscript PCOMPBIOL-D-23-01928

Dear Dr. Vico de Fallani,

We are happy to resubmit our manuscript entitled "Compression-based inference of network motif sets" for consideration as a methods article in PLOS Computational Biology.

Following the comments and recommendations of the two referees, we have substantially shortened the main body of the manuscript to make it more accessible. In order to focus on our methodology, we have in particular moved the description of the base and reference models to the supplement and replaced them by a new table (Table 1) that summarizes the expressions. In the same spirit, we have shortened the introduction to focus on the novel aspects of our method. Prompted by the referees, we have furthermore added additional tests and extended the discussion of our method's computational complexity, in particular how it may be applied without the need for high performance computing infrastructure.

We believe these revisions address the concerns of the referees and make the manuscript more accessible to the readership of PLOS Computational Biology. We address below the comments of the referees in a point-by-point manner.

We look forward to hearing from you,
Best regards,

Christian Vestergaard

Reviewer #1:

The authors present an inference method to identify motifs in graphs using the minimum description length principle for model selection.
I think that this is a nice contribution and is a good fit for PLoS Computation Biology.
I think that the authors do a good job of testing their approach in different scenarios, and also of considering a wide range of null models to make 'real' model selection. Overall, I think that this is a very robust approach to the problem of motif identification that should be adopted by the scientific community.

We thank the referee for having taken the time to read our manuscript and for their helpful comments. We have taken them all into account in our revised manuscript. We believe this has helped to make the paper more accessible to the readership of PLOS Computational Biology.

I have a few suggestions/comments that the authors could consider to improve their manuscript:

- Writing. Overall, I think that the manuscript is well-written but is somewhat dense. I fully understand this is a methodological paper (and I am all for it). However, for the general audience it might be a little bit too much to have to go through all the derivations to get to the plots they can understand. I would consider shortening some parts --- for instance, the expressions for the description lengths of the null models have already been derived somewhere else, so maybe these explanations are not really necessary and a table/list is enough? This is just a suggestion. I understand that the authors might want to keep consistency above all, but the paper is long.

Thank you for these suggestions. The paper is indeed quite long and technical and we had already devoted a lot of thought to making it as accessible as possible in the original version. Nevertheless, we agree that the derivations of the different codelengths for the base codes and reference (null) models were not necessary to understand our method. We have thus considerably shortened these sections and have replaced the equations in the main text by a table recapitulating the models' codelengths (Table 1 in the present manuscript). Prompted by this comment and similar ones by the other referee, we have also considerably shortened the Introduction section by refocusing it on the advantages of our approach and moving the discussion of the complications of hypothesis testing-based motif mining to the supplement. We believe these changes have made the manuscript more accessible to the reader.

- Algorithm and degeneracy. If I understand correctly each node in the original network can only belong to one motif which corresponds to the one motif that compresses the network the most. Are there ever ties, i.e. can a node belong to two motifs that are equally compressive? What happens then? This also leads to the question, is there a unique set of motifs that compresses the network the most, or are there several similarly compressive options (i.e. if I run the algorithm twice, do I get the same answer?). One would expect some level of degeneracy due to errors in connectome reconstruction. Then, can one really assign a set of motifs to a specific connectome without taking into account other compressive sets of motifs?

Due to the enormous number of possible configurations, we are not guaranteed to find the optimum motif set that compresses the network the most among all possible motif sets. This is why we rely on a stochastic greedy iterative algorithm to find a locally optimal motif set. To test the robustness of the procedure, we perform a hundred independent runs for each inference (for each base model and dataset). There are no guarantees that a significantly more compressing set does not exist, but as Figure 2D shows, the distributions of the codelengths are very peaked close to the minimal codelengths (we observe the same results for all connectomes).

Nevertheless, we stress that what we provide is, above all, a robust, safe statistical inference method that allows the selection of higher-order substructures (which is not possible using existing testing-based methods), and where correlations between them are permitted. While it would be tempting to keep motif sets from all algorithmic runs, to do so rigorously would require the development of a Monte Carlo (MCMC) sampling procedure (in order to evaluate the entropic contribution to the posterior distribution over motif sets), which is beyond the scope of the present manuscript. Out of all configurations, constituting a finite set of models encoding the same network, the MDL principle, as a model selection principle, singles out the one with the shortest description length as the best model for a biological network.

We have added the following paragraph to the discussion section discussing this challenge:

"Note that while our method enables statistically grounded inference of motif sets, it does not provide an estimate of their intrinsic statistical variability since it relies on a greedy optimization algorithm---in the language of Bayesian inference, inferred motif sets correspond to maximum a posteriori (MAP) estimates. This variability could in principle be estimated via Markov chain Monte Carlo (MCMC) sampling around the optimum motif set, but the development of an efficient MCMC algorithm is an open problem. Thus, for the time being, the variability can only be assessed experimentally by comparing multiple measurements."

INSTITUT PASTEUR

- Motif size. I imagine that there is a limitation to the size of the motifs one can consider at a computational level, but it was not clear to me what is the threshold value the authors used for that parameter. Is it possible to come up with an estimate of the largest motif that it would make sense to consider (given for instance the number of nodes in the networks and its density)? Intuitively, the larger the motif the more costly to describe, but also the most potentially compressive, so is there some size beyond which description cost outweighs compressiveness?

The practical limits on the size of motifs one can consider are mainly computational. The subgraph census is known to scale poorly with graphlet size, and if we have no a priori on the candidate graphlets their number grows super-exponentially with their size (for 3–5 node motifs we have 10k candidates, for 6 nodes more than 1M), which drastically increases computational cost and will also statistically favour models with less different graphlets. However, if we know which motifs to look for, we do not expect any a priori no theoretical limits with respect to the graphlet size. The cost of contracting a subgraph is not simply related to its size, but mainly to its (a)symmetry and its connections to the other (super)nodes of the graph.

- Motif size and density. The authors show in Fig 3 that for 300 nodes and rho=0.1 'low density' motifs are typically undetectable, and high density motifs (i.e. cliques) are detectable. Then, in Fig. 6 the authors show that the recurrent motifs across networks are, in fact, cliques. This makes me wonder, is this because of an existing 'detectability threshold' so that these dense motifs are the only motifs that are detectable (as Fig 5 also suggests)? This is connected to my previous question of wether there is a theoretical limit to the density of motifs that one can detect. Intuitively, cliques are cheap to describe but medium density motifs are more costly and therefor never selected. Is my intuition correct?

More symmetric motifs are indeed less costly to encode but we found empirically that the cost for encoding a subgraph contraction is in general largely dominated by the rewiring cost (the last term in Eq. (8)). When selecting between a clique and a less dense and less symmetric graphlet, the clique will indeed tend to be favored (not only due to symmetry but also since it removes more edges from H), but it also depends on the subgraphs' neighborhood. Thus, if dense subgraphs exist in a network we expect our method to select these over less dense ones since they are the most significant substructures (in the precise sense that they allow to compress the network more) and thus provide the better generative model for the network. The fact that we find a large amount of dense motifs in empirical connectomes means that these are the most significant structural patterns (amongst those we considered) from a statistical point of view.

If no significant dense subgraphs are present in a network our method will not detect them, as witnessed by our tests on numerically generated networks. Conversely, our approach can correctly detect motifs that are much less dense than cliques (Fig. 3 and Supplementary Figs. 3–6). Thus, if one looks specifically for sparser subgraphs (e.g. by excluding cliques from the possible motif set or by otherwise restricting the graphlet set using prior information), our method can correctly detect them.

- Limitations. Form what the authors said, users need a supercluster to get these results. Can they comment on how this limits the applicability of their approach?

A similar point was also raised by Referee #2 (see also our response below).

We indeed performed the subgraph census and motif-based inference on the HPC cluster of Institut Pasteur. As our method relies on listing all or at least a large amount of 3–-5-node induced subgraphs (cf. “Subgraph census” in the section “Material and methods” & Supplementary Note C), there is, for sure, a strong computational constraint. However, we have now added in the Supplementary Material (Supplementary Note B) the time and memory costs for two use cases: when the computational resources are limited (Table S1), versus our HPC-cluster support, (Table S2). We demonstrate that, for small-sized connectomes—typically, less than a thousand neurons—our algorithm can be run locally, on a common laptop (Supplementary Note B). This is especially true when the maximal graphlet size is reduced to 4, reducing the number of candidate motifs from approximately 10 000 to 200. In this context, which is the only context that is accessible using previous methods, the motif-based representation still surpasses the motif-free baselines. Also, the stochastic version of the subgraph census offers a great flexibility in terms of time complexity and memory costs, offering the possibility to adapt our method to computational resources available.

Minor edit:
If I understand correctly lines 211-212 are a retell of line 206.

Thanks for catching this! We have corrected it.

Reviewer #2:

In the manuscript "Compression-based inference of network motif sets", A. Benichou and colleagues propose a framework for motif mining based on lossless network compression using subgraph contractions. They applied this framework to synthetic benchmark and neural connectomes, which allow the evaluation of the collective significance of motif sets in terms of a null model inferred from the data.
Altogether, this represents a valuable contribution to the network and neuroscientific communities. However, certain sections need rephrasing and clarification before acceptance. Below are my comments and suggestions:

We thank the referee for their careful reading of our manuscript and for their useful comments. We have taken them all into account in our revised manuscript, which we believe has made our manuscript more accessible to the reader.

- While most of the manuscript is well-written, the abstract, introduction and part of the method could benefit from being more concise.

While we had devoted a lot of thought to making the paper as accessible as possible in the original version, we do agree that the paper is quite long and technical. The first referee also raised a similar point (see also our response above).

To make our manuscript easier on the reader, we have considerably shortened the Introduction section by refocusing it on the advantages of our approach and have moved the discussion of the complications of hypothesis testing-based motif mining to the supplement. We have furthermore moved the derivations of the different codelengths for the base codes and null models to the Supplement since they were not necessary to understand our method. We have furthermore replaced the derivation and equations for the base and reference (null) models in the main text by a table recapitulating their codelengths. This has considerably shortened these sections and, we believe, have made the manuscript more accessible to the reader.

- Also, in the introduction and abstract, explicitly mentioning that the approach was first assessed on numerical benchmarks and then applied to neural connectomes would enhance clarity.

Thank you for this suggestion. We have added the following sentence to the last paragraph of the introduction:

"We first validate our approach on numerically generated networks with known absence or presence of motifs."

- I believe that it would be important to expand on the time and space complexity of the proposed approach, including information about some of the cases reported in the manuscript (e.g. the authors mention the need of storing 1.3 TB of data for the Drosophila connectome). This will offer valuable insights into usability and complexity in practical cases.

Referee #1 raised a similar point about the computational complexity of our approach and the need for a HPC cluster to obtain the results presented in the manuscript (see above).

We wanted to showcase the limit of what could be obtained using our method by extending motif inference to 5-node motifs (of which there are approximated 10 000 candidates) from the computationally simpler case of 4-node motifs (of which there are approximately 200 candidates), which had previously been considered using hypothesis testing, and applying it to the most recent (and largest) synapse-resolution neural connectomes of biological neural networks available.

In this context, we indeed needed to perform the subgraph census and motif-based inference on the HPC cluster of Institut Pasteur to perform all repetitions and tests of our inferences. We have now added in the Supplementary Material (Supplementary Note B) the time and memory costs for two use cases: when the computational resources are limited (Table S1), versus our HPC-cluster support, (Table S2). We demonstrate that, for small-sized connectomes—typically, less than a thousand neurons—our algorithm can be run locally, on a common laptop (Supplementary Note B). This is especially true when the maximal graphlet size is reduced to 4. In this context the motif-based representation still surpasses the motif-free baselines. Also, the stochastic version of the subgraph census offers a great flexibility in terms of time complexity and memory costs, offering the possibility to adapt our method to computational resources available.

- Although the code and scripts used in the manuscript are available on GitHub, providing additional documentation and example cases on how to use the code and perform simple analyses would increase its usability among scientists working on these topics.

We thank the referee for the suggestion. We have added documentation in our GitLab project to guide through the main steps of running the motif-based inference. We also included the raw

and processed datasets, which gathers all the presently known completely mapped synapse-resolution connectomes. In the Supplementary Material (Supplementary Note B), we now additionally describe two categorical use-cases, depending on the user's resources, serving as an additional support.

- Even though the approach does not allow for overlapping contractions, the motifs identified in the C.elegans connectome could be also linked to recent works on symmetric motifs in C.elegans (e.g. www.nature.com/articles/s41467-019-12675-8). Are there any commonalities in the motifs obtained in the manuscript and the symmetric building blocks reported in the paper? If so, this could be useful to strengthen the importance of some of motifs identified in the empirical datasets.

Thank you for the suggestion. While we rely on similar concepts related to graph symmetries our paper considers local structures (subgraphs and their symmetries) whereas ***www.nature.com/articles/s41467-019-12675-8*** consider more global symmetries of a network. It seems like an important way forward for network analysis to include the concepts of symmetry groups of a network in an inferential framework or to consider interactions between local (such as motifs) and global symmetries. We have added a discussion of future directions of research in the inference of higher-order network structures to the Discussion section.

- In addition to the recent work on bayesian stochastic block modeling, the authors might consider exploring parallels with the hierarchical block model discussed in another study (journals.aps.org/prx/abstract/10.1103/PhysRevX.4).

The HSBM paper by Tiago Peixoto is indeed an inspiration for our methodology. We did not cite this paper initially since his later paper relying on a microcanonical description (Ref. 98) is more directly linked to our formalism. However, we agree that there are important parallels that might inspire future developments in the inference of higher-order network structures such as motifs. We have added discussions of this paper, both in the context of including hierarchical SBMs as base models for motif inference and as a possible inspiration for the definition of hierarchical motifs.

Minor Comments:

- Caption Figure 1: "In this example," instead of "In this eample,"

- Figure 2D: Adding a legend explaining the meaning of the colors would improve interpretation.

- Line 210: Correcting "m_{max.}" to "m_{max}"

- Lines 211-213: The paragraph appears to be redundant or repeated.

- Figure labels in Figure 3: Placing them on the side of the figure, as done in other figures, would be ideal.

Thanks for catching these! We have corrected them.
